# Diuretics

**Published:** 1910-12

**Authors:** George L. Servoss

**Affiliations:** Fairview, Nevada


For Texas Medical Journal.

### Diuretics.

BY GEORGE L. SERVOSS, M. D., FAIRVIEW, NEVADA.

Diuresis may be obtained by direct stimulation of the kidney functions, or secondarily through stimulation of other functions, which in turn force additional work upon the kidneys. There are numerous indications for enforced diuresis, as it is through this channel of elimination that we are able to rid the economy of many stored-up toxins. In conditions where we find an accumulation of fluids, as in anasarca, forced elimination of the fluids, by way of the kidneys, is the prime indication in order that relief may obtain. In many of the organic and functional diseases of the heart, the diuretics are indicated. In all conditions shown to be due to toxemia or acidemia, free elimination through the kidneys should be established and sustained until such time as the symptoms are relieved and a normal equilibrium established. It will be found that diuretics will, in many instances, act as direct synergists to other eliminants, and they should be considered in all cases where it is desirable that waste material be removed, as it is a recognized fact that the kidneys act with great efficiency in the removal of such material.

As is the case with all other remedial agents, full consideration should be given to all existent conditions prior to the use of any one diuretic agent, as the different indications require different remedies. In toxemia and acidemia, due to stored-up toxins, an agent acting directly upon the nephritic function would be indicated, while in anasarca due to faulty heart function, the agent to be employed would be one acting primarily upon the heart and secondarily upon the kidney, bringing about diuresis by raising of the blood pressure and thus enforcing the kidneys to carry off more of the fluid blood content. Elimination would follow in either case, but different in character. In acidemia, we have a chemical process with which to contend, while in anasarca the trouble is largely due to faulty function, and there is a call for a remedy which will bring about normal conditions, rather than for one which will remove chemical products. Consequently, it is plainly shown that diuretic agents should be chosen with care, and absolutely according to indications in each individual case.

Some agents act to increase the flow of fluids without causing an increase in the excretion of waste, while others increase the excretion of such material without a vast increase in the fluids. Both of these classes of agents are considered under the general heading of diuretics, and their various actions should be borne well in mind at all times when eliminants of this sort are under consideration.

It is not the purpose of this paper to take into consideration all of the known diuretics; only those which are the active principles of vegetable or plant life being discussed. There are many other agents applicable in this way, but they are well known and need no attention at this time.

Among the active principle diuretics we find arbutin, asparagin, boldine, barosmin, chimophilin, colchicine, collinsonin, digitalin, digitonin, eupurpurin, populin, scillitin, sparteine, strophanthin, and xanthoxylin.

Arbutin is a glucose from uva ursi. It acts as an astringent, coagulating albumin and constringing the cells, but not the blood-vessels (Brunton). In grain doses, Hughes found arbutin to be a powerful diuretic. It diminishes the exudation of albumin through the Malpighian tufts, and in this direction is said to be more powerful than is tannic acid. The specific indication is atony or hypersecretion of the mucosa. It combats putridity and sepsis and corrects putrid fermentation of the urine, and consequently is of value in all septic conditions, as cystitis, pyelitis, pyelo-nephritis and gonorrhea. It has been found useful in cystitis associated with enlarged prostate. Arbutin is suggested as a valuable remedy in all instances of acidemia with faulty elmination and as a diuretic in dropsy, and in other cases where prompt removal of fluid is required. Arbutin has other actions than that of a diuretic nature and is an agent worthy of close study and application. The dose is from 1-6 to 1 grain, and may be pushed without fear of untoward results.

While asparagin is credited with some diuretic power, this supposition has not been thoroughly established. It is excreted by the kidneys and gives the peculiar asparagus odor to the urine. It is probable that asparagin increases the excretion of solids by the kidney. as it has been found effective in gouty and rheumatic conditions. As diuretics, as such, are agents employed to reduce dropsical effusions, as a rule, and as asparagin does not have this action, to an appreciable extent, it can not, consequently, be classed as a true diuretic.

Barosmin is a resin obtained from buchu and has much the same action as has the entire plant. The actions of barosmin are so similar to arbutin that, as a rule, the latter meets all the indications, thus obviating the necessity of the employment of two agents to meet the same indications.

While boldine, the alkaloid from Peumus boldo, may not be considered as a true diuretic, it is to be considered under this heading, in that it has a pronounced action as an eliminant of solids by way of the kidney and is a synergist to the action of the more marked diuretics. Boldine not only increases the elimination of urea, but increases the flow of bile, and thus acts doubly as an eliminant. The indications for the employment of this agent are evident and need not be discussed at this time. It should be borne in mind that it is not a true diuretic and that when it is employed, with the idea of nephritic elimination, other agents should be employed in conjunction, in that the waste material may be promptly removed. The dose is 1-67 grain, the daily dose being from 6-67 to 10-67 grain.

The Eclectics consider Chimaphila umbellata, or pipsissewa, a very valuable diuretic, and it has been found that chimaphilin, a concentration of the entire drug, is equally active. It increases waste and effects nutrition, and the Eclectics have used it with benefit in scrofula, chronic rheumatism, irritations of the genito-urinary tract, genital discharges and other abnormal conditions of the genito-urinary apparatus. It will be found, on comparison, that arbutin is as valuable, as a diuretic, and that we have no need of consideration of other than the latter in this connection. The dose of chimaphilin is one grain before meals and at bedtime.

While small doses of colchicine act to increase the urinary flow, it is not, as a rule, employed as a diuretic, *per se,* but rather as a synergist to such action. It is a marked eliminant and may be employed with good results whenever such an agent is indicated and is mentioned in passing to bring out its importance in this direction.

Collinsonia canadensis, or stone-root, is a remedy advised by the Eclectics, acting as a general tonic and alterative, and as a diuretic. Collinsonin, the concentration of the entire plant, has much the same action as has the whole drug. It is recommended, as a diuretic, in cystitis, in which it is said to produce good results. It is also suggested in many other pathologic conditions, not under discussion at this time. The dose is 1-6 grain three or four times a day.

The diuretic action of digitalis is so well known as not to require discussion at this time. The active principles, digitalin and digitonin, both give the same diuretic action as does the whole drug. Digitalin, by stimulating the heart action, increases the flow of blood to the kidney, thus acting indirectly as a diuretic, while digitonin acts primarily as a diuretic, without cardiac stimulation to any appreciable extent. These agents are extremely valuable in all dropsical affections where the entire drug is indicated and are superior to the latter, in that they are always of a certain standard strength and, furthermore, that it has been found that cumulation is not liable to follow their exhibition. The dose of digitalin is 1-67 grain, and that of digitonin 1-500 grain.

Eupurpurin is a concentration from Eupatorium purpureum. The entire plant products are considered, by the Eclectics, to possess diuretic powers, and Lloyd says that the specific action is upon the kidneys, increasing both the solid and liquid constituents of the urine, and recommends it in renal dropsy. The special indications given are vesical irritation, incontinance of urine, painful and frequent micturition, urine scanty and milky with mucus or blood, uric acid diathesis, pain and weight in loins extending to the bladder. Eupurpurin gives the same action as do preparations of the entire drug and the dose is from 1-6 to 1 grain three or four times a day.

While populin, concentration from white poplar, is considered generally as a tonic and febrifuge, it has some diuretic action and has been found valuable in the relief of tenesmic vesical irritation and of tenesmus after urination. The dose is from 1-6 to 1 grain before meals and at bedtime.

Scillitin, sparteine and strophanthin have much the same action as have the active principles of digitalis and in some cases may be employed when the digitalis principles are, for one reason or another, contraindicated. These agents are worthy of study and application.

While xanthoxylin is not a direct diuretic, it will be found that it acts as a marked synergist to the action of other and direct diuretic agents, in that it is a marked eliminant and a remedy of great value in acidemia. The dose is 1-6 grain every hour or two to effect, and then at longer intervals to sustain such effect.

---

WE REGRET TO ANNOUNCE the death of Mrs. Annie P. Nichols November 20th, wife of Dr. J. R. Nichols, of Greenville, Superintendent Southwestern Insane Asylum, San Antonio, Texas. We extend our sympathy to the doctor.

---

DR. T. J. BENNETT, of Austin, has just returned from Chicago, where he took a post-graduate special at the great hospital and post-graduate schools.

